# Chlorine Dioxide: Antiviral That Reduces the Spread of ToBRFV in Tomato (*Solanum lycopersicum* L.) Plants

**DOI:** 10.3390/v16101510

**Published:** 2024-09-24

**Authors:** Ubilfrido Vásquez Gutiérrez, Gustavo Alberto Frías Treviño, Juan Carlos Delgado Ortiz, Luis Alberto Aguirre Uribe, Alberto Flores Olivas, Mariana Beltrán Beache, Francisco Daniel Hernández Castillo

**Affiliations:** 1 Parasitología Agrícola, Universidad Autónoma Agraria Antonio Narro, Calzada Antonio Narro, Saltillo 25315, Mexico; vagu.99@hotmail.com (U.V.G.);; 2 Consejo Nacional de Humanidades, Ciencia y Tecnología, Ciudad de México 03940, Mexico; 3 Centro de Ciencias Agronómicas, Departamento de Agronomía, Universidad Autónoma de Aguascalientes Posta Zootécnica, Jesús María, Aguascalientes 20700, Mexico

**Keywords:** chlorine dioxide, effectiveness, necrotic local lesion, phytotoxicity, transmission

## Abstract

Tomato brown rugose fruit virus (ToBRFV), being a mechanically transmitted disease, is usually difficult to control; therefore, an effective alternative to reduce transmission and replication in the crop is by spraying with chlorine dioxide (ClO_2_) during routine crop management. In this research, the efficacy of chlorine dioxide (ClO_2_) for ToBRFV management in a greenhouse and open field was determined. The phytotoxicity of ClO_2_ and its effective concentration against ToBRFV in *Nicotiana longiflora* plants were evaluated. Subsequently, the effect of ClO_2_ on ToBRFV was evaluated in tomato plants grown in an open field. Finally, the effectiveness of ClO_2_ on plants inoculated with ToBRFV under greenhouse conditions was evaluated and the number of necrotic local lesions (NLLs) was quantified. The results revealed that ClO_2_ at 760 mg L^−1^ did not show phytotoxicity and reduced the number of NLLs in *N. longiflora* plants. It also decreased ToBRFV transmission and replication in field- and greenhouse-grown tomato plants, improving agronomic parameters. ClO_2_ reduced replication in plants inoculated with different amounts of ToBRFV inoculum in a greenhouse. *N. longiflora* leaves expressed lower numbers of NLLs when inoculated with ClO_2_-treated tomato plant extracts. Finally, the results demonstrate that ClO_2_ represents an effective management alternative when used by direct application to plants. To our knowledge, this is the first study where the use of an antiviral compound is carried out under field and greenhouse conditions.

## 1. Introduction

Tomato (*Solanum lycopersicum* L.) is one of the most important vegetables at national and international level, due to its nutritional value for human consumption [1]. In Mexico, the states with the highest production are located in the Central and Northwest regions of the country, with an increasing production of 3,401,335 Ton Ha^−1^ [2]. One of the limitations that affect the production of this crop are systemic pathogens, specifically viruses that are transmitted mechanically and through seed miles [3]. The genus *Tobamovirus* involves a group of viral species with importance for agriculture, due to their genetic diversity, transmission mechanisms, adaptation, interaction, host range and limited antiviral chemical compounds [4]. The tomato brown rugose fruit virus (ToBRFV) species (*Tobamovirus fructirugosum*) was first detected in Jordan in 2015, and is currently considered a threat to tomato production worldwide [5]. Controlling the inoculum source and insect vectors could help in the management of plant viral diseases; however, the most effective approach remains plant genetic resistance [6]. Chlorine dioxide (ClO_2_) is a strong oxidizing agent recognized as a disinfectant with anti-nano-microbial activity capable of inhibiting virus replication in animals and humans [7]. ClO_2_ has long been applied as a biocide, for the disinfection of drinking water, for vector insects that are mostly reservoirs of phytopathogenic microorganisms and as human pathogens, qualities that could be identified as potential vehicles of epidemic disease outbreaks [8]. ClO_2_ has been used for post-harvest food preservation and to control bacteria such as *Escherichia coli* and *Listeria monocytogenes* and toxigenic fungi such as *Aspergillus flavus* and *Nosema bobycis* [9]. It has also been used to control fungi, such as *Botrytis cinerea* in green bell pepper, where the application of 150 µmol L^−1^ of ClO_2_ inhibited spore germination and changed mycelial morphology [10]. ClO_2_ achieved greater popularity in the 1980s; however, during the recent pandemic caused by SARS-CoV-2 (*Betacoronavirus pandemicum*) in 2019, its use gained relevance, where its application as an oral treatment reduced infections in humans [11,12]. Recent studies suggest that the efficacy of control strategies based on the use of chemical compounds, mostly disinfectants, reduce the spread of ToBRFV in tomato plants [13,14]. Others have delved into the effectiveness of disinfectants with antiviral activity evaluated in combination with ToBRFV infective sap, such as Virocid (Cid lines, Spain), Virkon Aotol, China), Menno florades (Royal Brink-man, Spain), Clorox bleach solution (Clorox, Mexico), DanKlorix (Drogerie arena, Germany), Cl-TSP, KOH and Virex (Diversey, Mexico) [15,16,17,18,19,20]. Biorational compounds could be effective in mitigating the risk of ToBRFV, such as silane–phosphane formulations on polymeric films (propylene), silicon oxide, methanol, garlic extract, Pickering emulsions coated with cinnamon–silicon and lactoferra-mine [15,18,20,21]. None of these, however, have been directly established in crops under practical field conditions or demonstrated their effect during routine crop management. Ref. [22] revealed that ClO_2_ has not only been used as an antiviral compound but also has a positive effect on plant growth regulation, where the application of 10 μg L^−1^ of ClO_2_ induced the regeneration of roots, shoots, axillary buds, biomass, root number and root length. Ref. [7] determined that ClO_2_ could be associated with cytotoxicity and, in turn, with the production of reactive oxygen species (ROS). ROS possess special functions such as the modulation, cell signaling and induction of mito gene responses, while the oxidative properties of ClO_2_ react with nucleic acids, oxidizing viral RNA, which makes them useful for virus elimination [23]. This means that, at lower concentrations, ROS defend against infectious pathogens, whereas high concentrations of ROS can disrupt normal cellular function and promote irreversible damage against lipids, nucleic acids and cellular proteins. It is possible that the increase in ROS occurs as a consequence of cytotoxicity leading to the inactivation of viruses in the host [24]. The mechanisms of action have not been studied in depth, but most of them are based on the generation of oxidative stress and the production of reactive oxygen species (ROS), which ultimately cause cell damage and toxicity in plants [25].

Ref. [7] reported that chlorine dioxide (ClO_2_) decreases viral activity in tobacco mosaic virus (TMV), a Tobamovirus considered a secondary progenitor of ToBRFV [26]. Therefore, the objective of this research was to evaluate the effect of ClO_2_ on the transmission and replication of ToBRFV in tomato plants grown in the field and greenhouse and to adjust a predictive model of yield losses.

## 2. Materials and Methods

### 2.1. Source of Inoculum

Infected leaves with symptoms similar to those described by [26,27] were collected from tomato greenhouses from a production region in northern Coahuila, from hybrid tomato plants (*Solanum lycopersicum* L.) 172-300 (Yüksel Tohum, Antalya, Turkey) of 40 days of age. Samples were transferred to the laboratory at Universidad Autonoma Agraria Antonio Narro and preserved at (−10 ± 2 °C). FQ3 isolation was performed on 40-day-old hybrid tomato plants 172-300 (Yüksel Tohum, Antalya, Turkey). For this, a ToBRFV inoculum was prepared with symptomatic tissue from the collected samples and macerated (1:10, *w*/*v*) in a phosphate buffer solution (PBS) with pH 8 (0.01 M), and celite (Sigma, Darmstadt, HE, Alemania) was added as an abrasive, following the method cited by [28] with some modifications. With a swab impregnated with 100 µL of infectious sap, it was dispersed on the two apical leaflets of each plant. The isolated FQ3 inoculum source was kept and renewed three times on tomato plants of the same variety during the experiment.

#### Identification of ToBRFV by RT-PCR

The detection of ToBRFV was confirmed by RT-PCR, following the methodology described by [29]. The oligonucleotides were ToBRFV-F (5’-AACCAGAGTCTTCTTCCCTATACTCGGAA-3’) and ToBRFV-R (5’-CTCACCATCTCT-TAATAATCTCCT-3’), designed to amplify a 475 bp fragment. The resulting amplification products were spotted on a 2% agarose gel and sent to the laboratory (Biociencia, Monterrey, NL., Mexico) for Sanger dideoxy sequencing [29]. The sequences were analyzed and uploaded to the NCBI database.

### 2.2. Determination of Phytotoxicity and Effectiveness of Chlorine Dioxide on Nicotiana longiflora

*Nicotiana longiflora* (Cav.) plants were planted using coarse sphagnum peat, perlite and vermiculite in a 1:1:1 ratio. Transplanting was carried out 40 days after sowing in 15 × 16 cm polyethylene bags. Plants were fertilized three times a week with 25% Steiner solution [30]. A ToBRFV inoculum was prepared from plants belonging to the FQ3 isolate following the procedure mentioned above and 100 µL was inoculated at a concentration of 1:10 (*w*/*v*) on half leaves of *N. longiflora*, which was considered a replicate.

The chlorine dioxide (Sayula, Jal, México)) used was purchased from Oxiplant. The concentrations evaluated (100, 250, 500, 760, 1520, 3040 and 6080 mg L^−1^) were prepared in distilled water. The negative control (NC) was distilled water, and the positive control was infected sap at 1:10 (*w*/*v*) (PC). The treatments were applied 30 min after inoculation, spraying a volume of 5 mL, covering exactly half of the leaf. This was conducted with a completely randomized experimental design. The evaluation of phytotoxicity was carried out 10 days post inoculation (dpi), using a millimetric leaf where the affected area and the total area were measured, and the percentage of phytotoxicity was calculated with the equation: $Phytotoxicity\;(\%)\frac{Áffected\;area\;}{Total\;area}\times 100$. The number of local lesions (NLLs) expressed in half leaves of *Nicotiana longiflora* was also recorded for each treatment evaluated. An analysis of variance, a comparison of means with the Tukey test, with a significance level of *p* > 0.01, was carried out with the statistical program SAS version 9.1. (Cary, NC, EE.UU.)

### 2.3. Experiment under Open-Field Conditions

**Figure 1:**
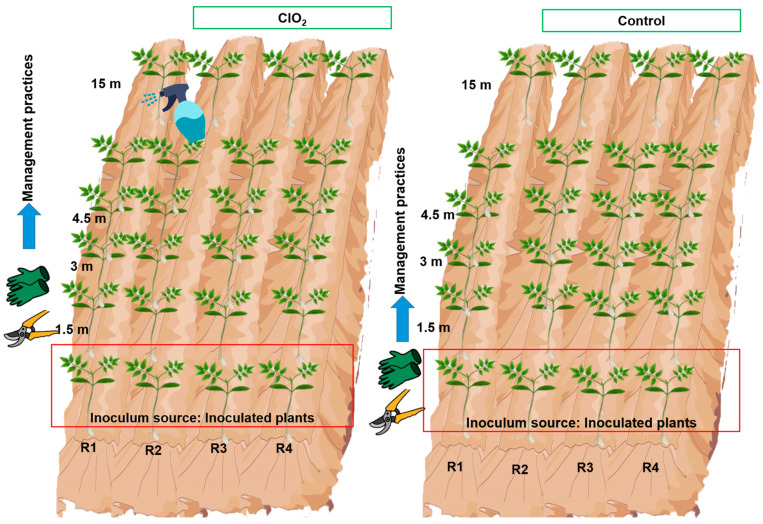
Paired open-field plots and chlorine dioxide spraying. Control: plants inoculated with infective sap but not treated; Row 1 to Row 4 of established plants.

Seeds of the hybrid genotype 172-300 F1 (Yüksel Tohum, Antalya, Turkey) were sown for this trial (Figure 1). Thirty days after sowing, the plants were budded to induce new apical shoots and transplanted under open-field conditions. Plants underwent fertigation with different percentages of Steiner solution [30] according to the phenological stage of the crop, plant emergence to vegetative growth (25%), vegetative growth (50%), flowering and fruit growth (75%) and during fruit filling and harvest (100%). The variables to be evaluated were: virus spread, area under the disease progress curve, agronomic variables and yield loss prediction. 

#### 2.3.1. Evaluation of the Effect of ClO_2_ on ToBRFV Propagation vs. Distance from the Inoculum Source

The trial started with inoculation of the inoculum source (IS) 15 days after transplanting, at the beginning of the four furrows per plot. A 1:10 (*w*/*v*) ToBRFV inoculum was prepared in phosphate buffer solution pH 8.0 (0.01 M). A total of 100 µL of infective extract was inoculated on the two first apical leaves of each plant and dispersed with a swab. When the plants located at the beginning of each furrow (IS) expressed the first symptoms (10 dpi), the application of treatments was started. ClO_2_ was prepared at 760 mg L^−1^ and plants located from the beginning of each furrow (IS) up to 15 m were sprayed; this procedure was performed for the four furrows (Figure 1, ClO_2_). Using a Pacto 320030 sprinkler (Swissmex, Saltillo, Coah, Mexico), the entire plant area was sprayed. The plant was handled 10 min after application. A total of 21 sprays were made from the growth phase of the crop, through development, to fruit ripening.

An experimental design of paired plots was established for the trial. The first plot consisted of plants sprayed with chlorine dioxide (ClO_2_) and the second plot consisted of unsprayed plants, considered as a control (Figure 1). For each plot, four 17 m furrows with 55 plants per furrow were evaluated. The inoculated plants at the beginning of each furrow were considered as the inoculum source (IS), and the treatments were the plants at distances of 1.5, 3, 4.5, 6, 7.5, 9, 10.5, 12, 13.5 and 15 m from the IS. Five plants per distance (treatment) were considered.

Confirmation of ToBRFV viral spread was performed by optical density (OD) in DAS-ELISA (Agdia, Elkhart, IN, EE.UU.) at 405 nm 50 days post inoculation (dpi). Plant samples were processed and collected from the beginning of each furrow up to 15 m. Samples were selected from the first apical leaflet of each plant and were taken at intervals of 1.5 m in length. This applied for each of the four 17 m furrows. The procedure was repeated for both ClO_2_-sprayed and unsprayed plants. Plants considered as the inoculum source, located at the beginning of each furrow, were used as a positive control (PC) (they were not added in the results). For plate sensitization, capture antibody (ACC 00960) (Agdia, Elkhart, IN, EE.UU.) was added at a concentration of 1:200 µL in Carbonate Coating Buffer (CCB) 1X and incubated (12 h at 6 ± 2 °C) under refrigeration. Subsequently, symptomatic tissue was macerated at a concentration of 1:10 (*w*/*v*) in extraction bags (12 × 15 mm) (BioCiencia, Monterrey, NL, Mexico) with General Extract Buffer (GEB) 1X (pH 7.5), the plate was washed three times with PBST Buffer (1X) and 100 µL of both the extracted samples and controls were added. The plate was incubated for 2 h at room temperature (22 ± 2 °C). The enzyme conjugate (ECA ACC 00960) (Agdia, Elkhart, IN, EE.UU.) was added at a concentration of 1:200 µL in ECI Buffer 1X (pH 7.5); the plate was washed eight times and incubated for 2 h at room temperature. Finally, PNP substrate was prepared in Buffer 1X (pH 9.8) at a concentration of 1 mg mL^−1^, and 100 µL was poured into each well of the plate. The optical density (OD) was measured at 405 nm using a Multiskan GO microplate spectrophotometer (Thermo Fisher^TM^, Alcobendas, M, Spain). A total of three readings were taken every 15 min. The viral concentration in the samples was measured by taking as a criterion the average of the absorbances of the treatments evaluated.

#### 2.3.2. Estimation of the Area under the Curve of ToBRFV Disease Progression

Severity measurement was performed at 10-day intervals up to 50 dpi, and the equation proposed by [31] was applied based on the diagrammatic scale of severity by [32]. With the data obtained, the area under the curve of disease progress produced by ToBRFV (AUCDP) was estimated, using the equation proposed by [33]:

$$AUCDP=\sum_{i=1}^{n}\;\left[\frac{Y_{i}+Y_{i+1}}{2}\right](+X_{i+1}-X_{i})$$

#### 2.3.3. Agronomic Variables

Measurement of variables was recorded according to crop development; at 30 dpi, variables of number of flowers (NFL), SPAD units and nitrogen were recorded. The number of fruits (NFR) and yield per plant were measured 50 dpi, three times a week for six weeks, and Tons Ha^−1^ were estimated. Brix degrees were measured at the end of the fruit harvest with a refractometer (Biotempak, Aguascalientes, Ags, Mexico) from the extract of 20 fruits per treatment. The variables were processed using a completely randomized block analysis with factorial arrangement, and the means were compared using Tukey’s test, with a significance level of *p* > 0.01.

#### 2.3.4. Predicting Tomato Yield Losses in the Open Field

Six epidemiological models were analyzed (Table 1). The best model was selected according to the slope of the line (*r*), coefficient of determination (*R*^2^) and the variation in the modeled data. The model will allow estimation of the losses produced by ToBRFV in tomato plants under open-field conditions using the viral concentration in plants measured by OD ELISA at 405 nm at 50 days post inoculation.

### 2.4. Experiment under Greenhouse Conditions

Plants of the 172-300 F1 hybrid genotype were used for this experiment. They were transplanted in 5 kg polyethylene bags using as substrate, coarse sphagnum peat, perlite and vermiculite in a 1:1:1 ratio. Plants were irrigated with different percentages of Steiner solution [30] according to the phenological stage of the crop, plant emergence to vegetative growth (25%), vegetative growth (50%), flowering and fruit growth (75%) and during fruit filling and harvest (100%). Viral propagation, AUCDP and agronomic variables were evaluated.

#### 2.4.1. Effect of ClO_2_ on the Propagation of ToBRFV vs. Viral Dilutions of the Inoculum

The experiment began with the preparation of five dilutions of infective sap (1 × 10^−3^ to 1 × 10^−5^) from an inoculum prepared at a ratio of 1:10 (*w*/*v*) in STF with pH 8.0 (0.01 M) and celite (Sigma, Darmstadt, HE, Alemania) as an abrasive. Subsequently, plants were inoculated as described in the previous experiment. This procedure was repeated for each treatment (dilution). ClO_2_ (Oxiplant, Sayula, Jal, Mexico) was prepared at a concentration of 760 mg L^−1^ in distilled water. One day post inoculation (dpi), ClO_2_ was sprayed on freshly inoculated tomato plants with different dilutions; the area of the plant was completely covered with a volume of 20 mL. A factorial design with two factors was established: As factor one, plants with and without ClO_2_ spraying. Factor two: the treatments evaluated were plants infected with different viral dilutions: T1 (1 × 10^−3^), T2 (1 × 10^−3.5^), T3 (1 × 10^−4^), T4 (1 × 10^−4.5^) and T5 (1 × 10^−5^). A healthy negative control (NC) was used, which were plants inoculated as PBS, and a positive control (PC) included plants inoculated with infective sap extract at a ratio of 1:10 (*w*/*v*) in PBS. There were 12 plants per treatment, where one tomato plant was considered as a replicate.

The spread of ToBRFV was measured from the viral concentration by optical density (OD), using the DAS-ELISA technique (Agdia, Elkhart, IN, EE.UU.) 50 dpi. The processed samples corresponded to three leaflets per replicate (plant) collected from the first vegetative shoot. The same arrangement was followed for the different viral dilutions (treatments). The DAS-ELISA technique was performed as described in a previous experiment. 

Variables were evaluated in a factorial design, using ANOVA. Posteriorly, means’ comparisons were performed using Tukey’s test, with a significance level of *p* > 0.01.

#### 2.4.2. Estimation of ABCPE by ToBRFV

Severity was measured at 10-day intervals up to 50 dpi, as mentioned in the previous experiment. With the data obtained, the area under the curve of disease progress produced by ToBRFV (AUCDP) was calculated, using the equation proposed by [33].

#### 2.4.3. Evaluation of Agronomic Variables

Variables’ measurements were recorded according to crop progress; 35 dpi the variables of number of flowers (NFL), stem diameter (cm) (SD) and plant height (cm) (PH) were recorded. At 70 dpi, the number of fruits (NFR), and yield (expressed in Ton Ha^−1^) for ToBRFV-infected plants sprayed with and without ClO_2_ were recorded. Fresh weight plant (FWP) and root fresh weight (RFW) were obtained with an analytical balance (Ohaus, Ciudad de México Mexico) and then dehydrated in a drying oven (Felisa TE-H80DM, Zapopan, Jal, Mexico) to obtain plant dry weight and root dry weight (PDW and RDW). The variables were processed using a randomized complete block analysis. Subsequently, mean comparisons were performed using Tukey’s test, with a significance level of *p* > 0.01.

## 3. Results

### 3.1. Identification of Inoculum Source ToBRFV

**Figure 2:**
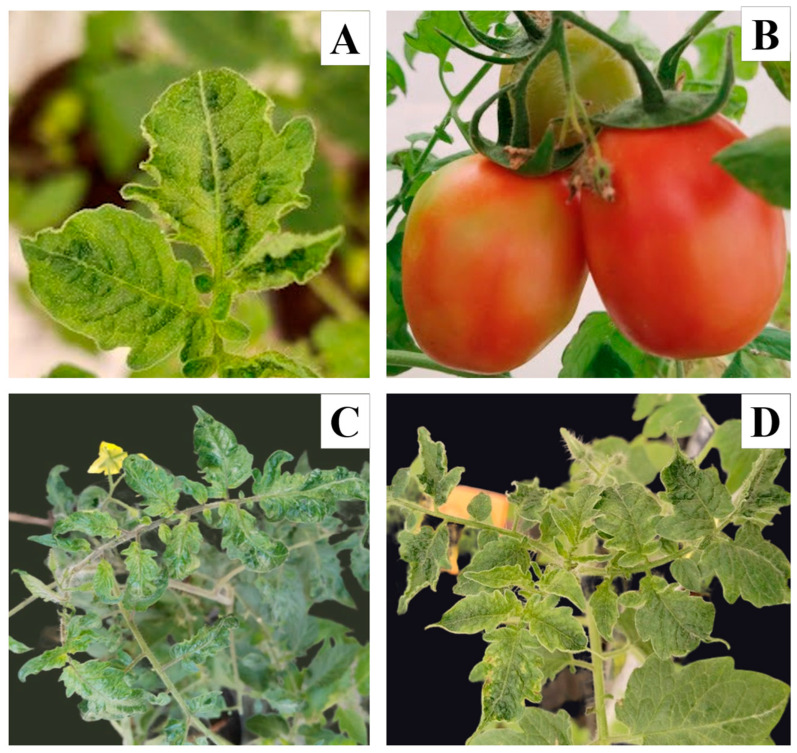
Leaf and fruit symptoms due to ToBRFV infection in tomato plants. (**A**) Blistering on leaves; (**B**) fruits with irregular ripening; (**C**,**D**) mosaic and severe yellowing.

Leaflets and fruits inspected from tomato plants belonging to ToBRFV inoculum source isolated FQ3, showed symptoms such as severe mosaic, interveinal yellowing, blistering, while in fruits: irregular ripening and mottling were seen similar to those reported by [26,27] (Figure 2). A 399 bp fragment (accession PP694587) was amplified with RT-PCR and was compared with the sequences registered in NCBI showing 100% similarity with accessions (OQ427353; KT383474; OK339579; OQ190155).

### 3.2. Phytotoxicity and Effectiveness of Chlorine Dioxide on Nicotiana longiflora

**Figure 3:**
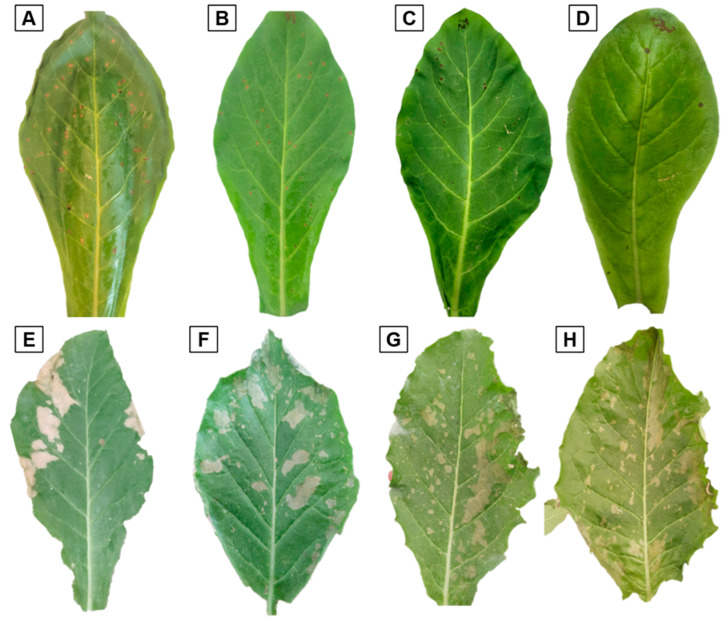
Effectiveness of chlorine dioxide against ToBRFV. (**A**) Positive control (1: 10, p/v); (**B**) 100 mg L^−1^ ClO_2_ + ToBRFV; (**C**) 500 mg L^−1^ ClO_2_ + ToBRFV; (**D**) 760 mg L^−1^ ClO_2_ + ToBRFV; (**E**) 1520 mg L^−1^ ClO_2_ + ToBRFV; (**F**) 3040 mg L^−1^ ClO_2_ + ToBRFV; (**G**) 6080 mg L^−1^ ClO_2_ + ToBRFV; (**H**) 6080 mg L^−1^ ClO_2_.

Results showed that 500 mg L^−1^ did not produce phytotoxicity in *Nicotiana longiflora* leaves but expressed a higher number of necrotic local lesions per milliliter (mL), which affected the ToBRFV viral load, while 3040 and 6080 mg L^−1^ totally reduced the appearance of NLL as an infection response to ToBRFV. ClO_2_ spraying caused (26.55) followed by (44.31) of phytotoxicity in *N. longiflora* plants (Figure 3G,H) compared to 760 mg L^−1^, which did not produce phytotoxicity and produced a lower amount of NLL per mL (15.30), which demonstrates its effectiveness (Figure 3C). ClO_2_ at a concentration of 760 mg L^−1^ turned out to be more effective in reducing the appearance of NLL by up to 95.98% (Table 2).

Chemical compounds have been studied so as to be used as disinfectants and antivirals that reduce the viral concentration of phytopathogenic viruses in greenhouse tomatoes [39]. Generally, to determine the effective concentration of a chemical against fungi and bacteria, the reduction in colony-forming vital units per milliliter in nutrient media is evaluated [40,41]. However, due to the need of a host cell for virus replication, such methodology cannot be developed for phytopathogenic viruses. Therefore, several authors [16,17,42,43] have studied the efficacy and phytotoxicity of disinfectants against ToBRFV with the use of *Nicotiana glutinosa*, *N. clevelandii, N. tabacum* cv. Xanthi NN and *N. longiflora* plants, comparing the measurement of the number of necrotic and chlorotic local lesions in inoculated and treated leaves with respect to untreated ones.

### 3.3. Open Field: Effect of ClO_2_ on the Spread of ToBRFV vs. Distance from the Inoculum Source

**Figure 4:**
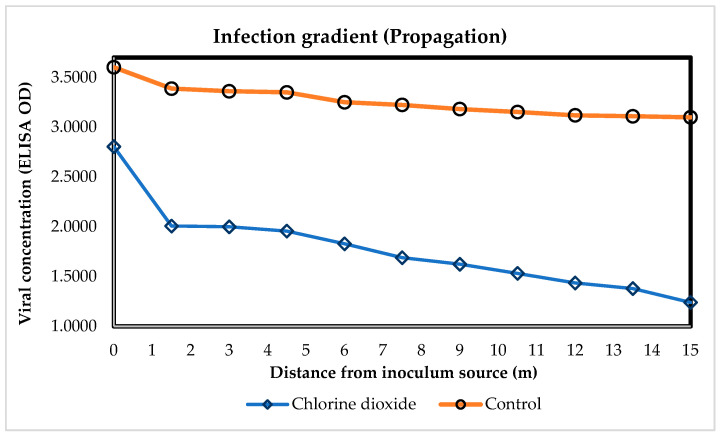
Gradient of ToBRFV propagation under the effect of chlorine dioxide on plants grown in the open field. ELISA OD: viral concentration measured by optical density; ClO_2_: plants sprayed with chlorine dioxide [y = 0.05 (d) − 2.16], R^2^ = 0.98342; Control: plants not sprayed with ClO_2_ [y = 0.02 (d) − 3.41], R^2^ = 0.95233. d: distance from the inoculum source.

A reduction in viral propagation measured by optical density (OD) was observed in relation to the distance of plants located at the beginning of each furrow, considered the inoculum source (IS); it was three times more pronounced in plants sprayed with ClO_2_ (Figure 4). In an epidemic, when the amount of initial disease (*y*_0_) approached 1.0, the inclination decreased to ”0”, commonly named the apparent infection rate (*r*) [44]. Results showed a reduction in the progress of the disease with a slope (*r* = −0.05), and an amount of initial disease (*y*_0_ = 2.16) in tomato plants treated with ClO_2_. Sprays with ClO_2_ reduced the concentration of ToBRFV in plants located at the beginning of the furrows (IS) by 22.14% with respect to untreated plants. Plants located at a distance of 1.5 m to 15 m expressed a decrease in viral spread from 44.21 to 65.58% with respect to the inoculum source. Plants that were not sprayed with ClO_2_ reflected a lower slope, which influenced the spread of the disease (−0.02), followed by the amount of disease transmitted among plants (*y*_0_ = 3.41), causing an almost straight inclination (0). Plants treated with ClO_2_ at 760 mg L^−1^ had a significantly decreased, by 60%, transmission of viral particles as a function of distance, at 50 dpi.

#### 3.3.1. AUCDP by ToBRFV in Tomato Plants Distant from the Inoculum Source

Severity determined as the area under the disease progression curve (AUCDP) by ToBRFV was reduced according to ClO_2_ sprays at 760 mg L^−1^, (Table 3). Plants located at the beginning of each furrow (IS) and sprayed with 760 mg L^−1^ ClO_2_, expressed a 16.95% reduction in the AUCDP with respect to untreated plants, whereas plants located from 1.5 to 15 m distant from the IS presented a decrease in severity from 72.62 to 85.34%, with respect to plants not treated with ClO_2_.

#### 3.3.2. Effect of ClO_2_ on Agronomic Variables of Tomato Plants

**Figure 5:**
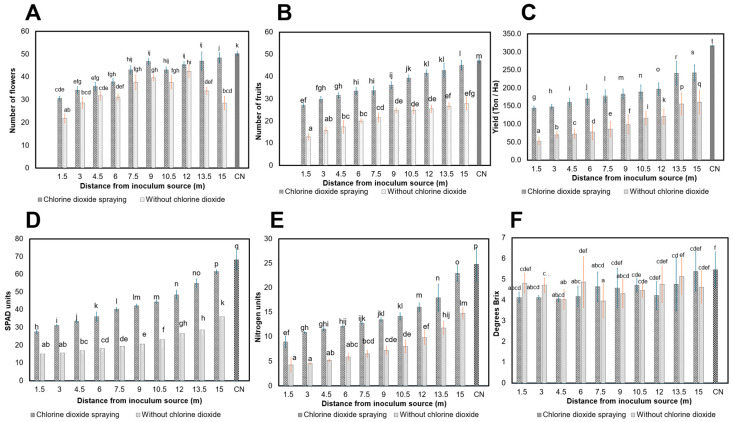
Effect of ToBRFV with chlorine dioxide sprays on tomatoes grown in the open air. (**A**) Number of flowers; (**B**) number of fruits; (**C**) yield (Ton Ha−1); (**D**) SPAD units; (**E**) nitrogen units; (**F**) degrees Brix. 1.5 to 15: distance from ToBRFV inoculum source. Means with a common letter are not significantly different (*p* > 0.01).

Field-grown tomato plants treated with ClO_2_ at 760 mg L^−1^ produced a positive effect on some parameters evaluated. Statistical difference with the ClO_2_ spraying was observed with respect to plants distant from the inoculum source (IS) (1.5 to 15 m) in terms of the number of flowers (NFL) (17.53 to 39.64%) (Figure 5A), compared to untreated plants (*p* < 0.0001). In the number of fruits (NFR), no statistical difference was found between distances, as well for plants that were not sprayed with ClO_2_ (*p* = 0.0232). The ClO_2_ produced a substantive effect at the distances of the IF (1.5 to 15 m), where the NFR increased (30.3 to 36.65%), compared to untreated plants. That is, the amount of fruits increased with ClO_2_ sprays with respect to the distance from the inoculum source (Figure 5B). The SPAD units representing the estimation of chlorophyll in the plant expressed a relationship with respect to the decrease in the propagation by ToBRFV (Figure 5D), in which plants located at the beginning of the furrows (IS) up to 15 m showed differences with the ClO_2_ spraying of 18.34 to 37.30%, compared to non-sprayed plants; differences between distances were observed but were not statistically different (*p*-value < 0.0014).

Nitrogen units showed statistical differences (*p* < 0.0001) 1.5 to 15 m from the inoculum source, where ClO_2_ sprays showed improvements of 18.8 to 32.86% compared with plants not infected with ToBRFV (Figure 5E). The brix degrees representing the sugar content in the fruits did not show statistical differences in fruits harvested from plants infected with ToBRFV (*p* > 0.0946), but there was a minimum difference of 2.37% with the ClO_2_ spraying, whereas fruits harvested from plants not infected with the virus showed values of 5.47 brix degrees (Figure 5F). Yield extrapolated to a planting density of 37,037 plants per hectare showed a significant increase in the plants sprayed with (*p* < 0.0001) (Figure 5C). That is, plants located at the beginning of the furrow (IS) showed a 45.7 Ton Ha^−1^ yield followed by the distances to the IS. (1.5 to 15 m) which showed a yield of 144.3 to 241.8 Ton Ha^−1^. Untreated tomato plants showed yields in the inoculum source (42.8 Ton Ha^−1^) and at distances (1.5 to 15 m) yielded 52.8 to 160.6 Ton Ha^−1^. On the other hand, plants not infected with ToBRFV showed a yield of 290 Ton Ha^−1^. Plants not sprayed with ClO_2_ showed yield losses ranging from 63.91 to 33.58% (1.5 to 15 m) of the IS with respect to treated plants. Spraying with ClO_2_ prevented yield losses of 26.44% with respect to the negative control. These results demonstrate that ClO_2_ at 760 mg L^−1^ not only reduces the spread and concentration of ToBRFV with respect to the distance from the inoculum source but also improves the agronomic parameters in Yüksel Y172-300 F1 hybrid tomato plants under field conditions.

#### 3.3.3. Estimation of Yield Losses of Plants Grown in the Open Air

**Figure 6:**
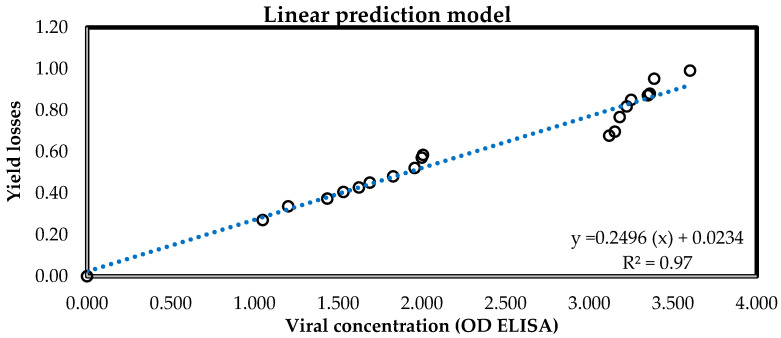
Adjustment of a linear model for the estimation of yield losses from the viral concentration in tomato plants grown in the open field. (x): viral concentration BY ELISA.

The model that showed the greatest adjustment according to the coefficient of determination (0.97) was the linear model proposed by [38]. (Table 4, Figure 6), where the equation showed an apparent infection rate, or slope of the line (*r*) of 0.2497, with an initial disease amount ($y_{0}$) of 0.0234. Ref. [44] posited that when r increases to 0, i.e., decreases, $y_{0}$ increases and approaches 1. Our linear model explains the opposite. As r approaches “$y_{0}$” moves away from 1. It can be said that with regard to the slope of the *r* line, in this case, the yield losses increase in relation to the amount of initial disease (*y*_0_ = 0.0234) with respect to the viral concentration in the plant (X axis) at 50 days after the ToBRFV infection.

This estimation model allowed the prediction of yield losses according to the viral concentration in the plant. Fifty days after infection, apical leaflets were collected from tomato plants that were infected with ToBRFV 55 days after planting. The collected samples were processed by DAS-ELISA and measured using a Multiskan GO spectrophotometer, and according to the viral concentration (DO) and adjusted linear model, the yield losses were estimated.

### 3.4. Greenhouse: Effect of ClO_2_ on the Spread of ToBRFV with Respect to Infection by Viral Dilutions

**Figure 7:**
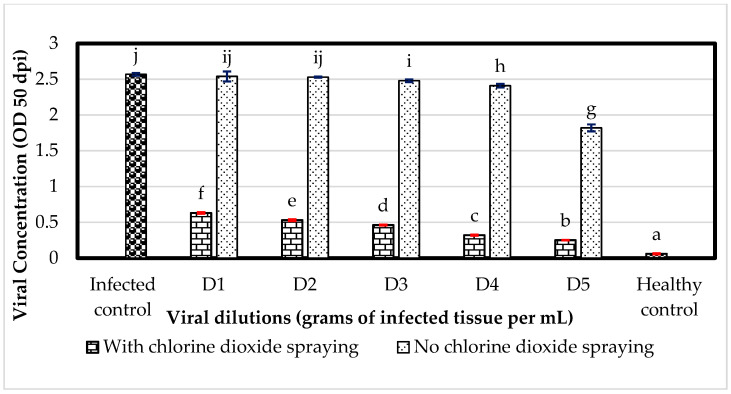
Effect of ClO_2_ application on viral dilutions of infected ToBRFV in tomato plants grown in greenhouse. OD: optical density based on viral concentration; dpi: days post inoculation; infected control: plants inoculated with 1 × 10^1^; plants infected with different viral dilutions of ToBRFV, D1: 1 × 10^−3^ to D5: 1 × 10^−5^; healthy control: plants inoculated with phosphate buffer solution (PBS). Means with the same letter are not significantly different (*p* > 0.01).

Plants grown in greenhouse conditions and inoculated with 1 × 10^−3^ without treatment (ClO_2_) expressed an increase in viral concentration (OD) of 75.19% compared to plants treated with 760 mg L^−1^ of ClO_2_, while the positive control (PC) showed (2.57, OD). Plants infected with 1 × 10^−5^ and sprayed with ClO_2_ reduced the viral concentration by 86.26%, unlike untreated plants. The PC infected with sabia prepared at 1 × 10^1^ expressed a viral concentration 1.16% above plants infected with 1 × 10^−3^ without treatment and 75.48% of treated plants. The treatments evaluated without ClO_2_ did not express statistical difference, but they did between OD values (Figure 7).

#### 3.4.1. AUCDP by ToBRFV

Results showed that the progress of ToBRFV in tomato plants was significantly reduced with the application of ClO_2_ (Table 5). The area under the disease progression curve (AUCDP) increased in relation to the progression of ToBRFV infection. Plants infected with viral dilutions from 1 × 10^−3^ to 1 × 10^−5^ and treated with ClO_2_ had reduced severity from 52.35 to 54.34% with respect to untreated plants. The AUCDP was reduced in plants infected with inoculum amounts of 1 × 10^−3^ to 1 × 10^−5^ (34.05 to 11.27%) compared to plants treated with ClO_2_ expressing a severity of 71.46 to 24.70% with respect to the positive control. These results show that the AUCDP gradually decreased with respect to the viral dilutions and the amount of viral particles in plants; on the other hand, ClO_2_ reduced the severity with respect to the viral load present in inoculated plants.

#### 3.4.2. Effect of ClO_2_ on Agronomic Parameters of Plants Grown in Greenhouse

**Figure 8:**
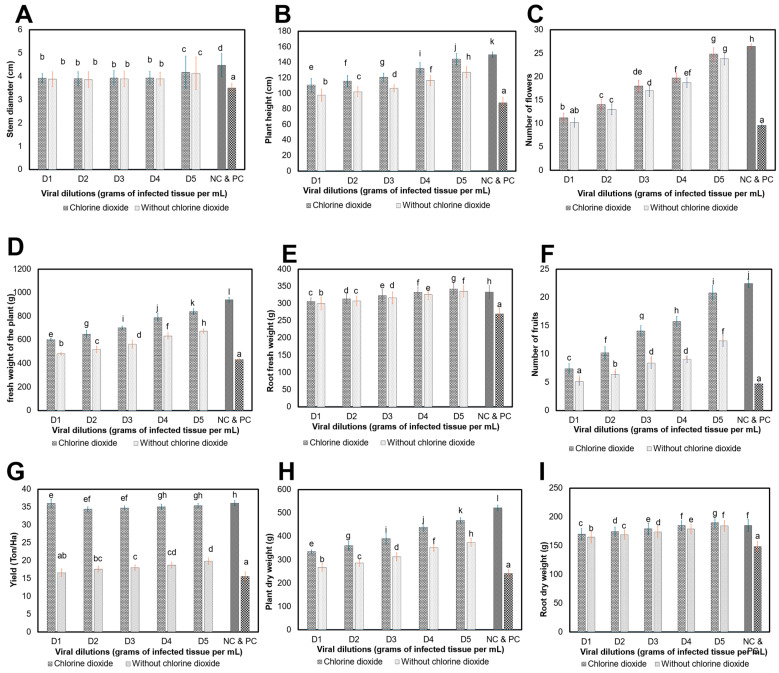
Effect of ToBRFV with chlorine dioxide sprays on tomato plants grown in greenhouse. (**A**) Stem diameter; (**B**) plant height; (**C**) number of flowers; (**D**) fresh weight of the plant; (**E**) root fresh weight; (**F**) number of fruits; (**G**) yield (Ton Ha^−1^); (**H**) plant dry weight; (**I**) root dry weight. D1: 1 × 10^−3^ to D5: 1 × 10^−5^. Means with the same letter are not significantly different (*p* > 0.01).

Results demonstrate that chlorine dioxide (ClO_2_) improved some agronomic variables (Figure 8). There was no statistical difference in the stem diameter (SD) (*p* < 0.9999); however, the ClO_2_ spray improved the SD by 2.49% (Figure 8A). In plant height (PH), a statistical difference was observed between treatments (viral dilutions) of untreated plants (*p* < 0.0001), compared to plants treated with ClO_2_, where 12.29% were improved (Figure 8B). The variable number of flowers (NFL) improved by 3.77% with ClO_2_ spray with respect to the infection of different viral dilutions of ToBRFV, whereas the negative and positive controls (NC and PC) presented values of 26.46 followed by 9.6, which means that the NFL was affected in 63.71% of plants with the ToBRFV infection (*p* > 0.9999) (Figure 8C). The fresh weight (FWP) and dry weight of the plant (PDW) showed significant statistical differences between the viral dilutions inoculated in plants, and also improved by 17.04% with the ClO_2_ spray in FWP (*p* < 0.0001), while the PDW was improved by 17.12% (*p* < 0.0001). On the other hand, the fresh root weight (RFW) increased by 1.89% (*p* < 0.2008) with ClO_2_ spray with respect to the viral dilutions present in tomato plants. On the other hand, the dry weight of roots (RDW) showed an improvement of 2.90% (*p* = 0.1987) (Figure 8D,E,H,I). Plants used as negative control presented values of 940.41, 522.45, 333.32 and 185.17 g according to the variables FWP, PDW, RFW and RDW, compared to the positive control with values of 433.66, 240.92, 270.04 and 148.27 g. Treatments evaluated showed a statistical difference in the number of fruits (NFR) (*p* < 0.0001), as compared to plants sprayed with chlorine dioxide. Spraying with ClO_2_ increased the NFR by 37.69% in plants infected with 1 × 10^−5^, compared to plants that did not receive treatment, whereas plants inoculated with 1 × 10^−3^ and sprayed with ClO_2_ only improved by 10.1%. Plants considered as NC and PC presented values of 22.47 and 4.73, which evidenced a decrease of 78.94% NFR before ToBRFV infection (Figure 8F). Yield extrapolated to Ton Ha^−1^ showed no statistical difference between treatments (*p* = 0.0254), but there was a difference in plants sprayed with ClO_2_ (Figure 8G). Plants infected with 1 × 10^−3^, 1 × 10^−4^ and 1 × 10^−5^ showed yields of 36, 34.73 and 35.41 Ton Ha^−1^, compared to untreated plants infected with the same amounts of inoculum, which presented 16.6, 18.01 and 19.82 Ton Ha^−1^. Application of ClO_2_ caused losses of 2.49% using a viral amount of 1 × 10^−5^ and plants had a 45.88% yield loss. Plants infected with 1 × 10^−3^ produced losses of 54.14%, while the ClO_2_ spray reduced the losses by 7.08%. There was no significant difference in the agronomic parameters of the crop due to the infection of the different inoculum amounts of ToBRFV, but it did affect the effect of ClO_2_ on the agronomic variables evaluated.

## 4. Discussion

Concern to investigate antiviral compounds that partially or totally inhibit the replication and transmission of ToBRFV is fundamental in the disease’s management; however, there are few studies using antiviral compounds. Mostly, in vitro evaluations with disinfectants and chemical compounds stand out but are limited under field and greenhouse conditions. The effectiveness of chlorinated trisodium phosphate (TSP-Cl) has also been highlighted with low percentages of soil-mediated ToBRFV infection. Refs. [19,40] used menno florades (active ingredient: benzoic acid) at 4% for the quantification of ToBRFV and to determine the disinfectant efficacy against the virus. Ref. [17] proposed the disinfectant menno florades as being capable of inactivating the virus on carpets, shoe soles, tools, iron and aluminum surfaces. Ref. [21] evaluated formula-two anti-biofilms from thin silane–phosphonium coatings on polymeric films (polypropylene) and SiO_2_ that showed antiviral efficacy, eliminating the appearance of necrotic lesions on tobacco leaves as a response of ToBRFV infection. The studies carried out count the number of local lesions and, in this way, evaluate the antiviral effectiveness between the treatments evaluated.

Inoculation on *Nicotiana tabacum* cv. Leaves using Xanthi-NC induced the appearance of necrotic local lesions (NLLs) by tobacco mosaic virus (TMV) (*Tobamovirus tabaci*) and were significantly reduced with increasing ClO_2_ concentrations. Results indicate that 760 mg L^−1^ expressed 1.53 NLLs in *N. longiflora* leaves, and did not cause phytotoxicity, which demonstrated the effectiveness against ToBRFV. NLLs appeared at 10 dpi and began coalescing and necrosing at 20 dpi. Reference [42] commented that the appearance of NLLs in leaves of *N. benthamiana* and *N. rustica* oscillate between four and six dpi; after this, they observed necrosis and NLLs that coalesced into systemic symptoms (severe chlorosis and mosaic) at 15 dpi.

**Figure 9:**
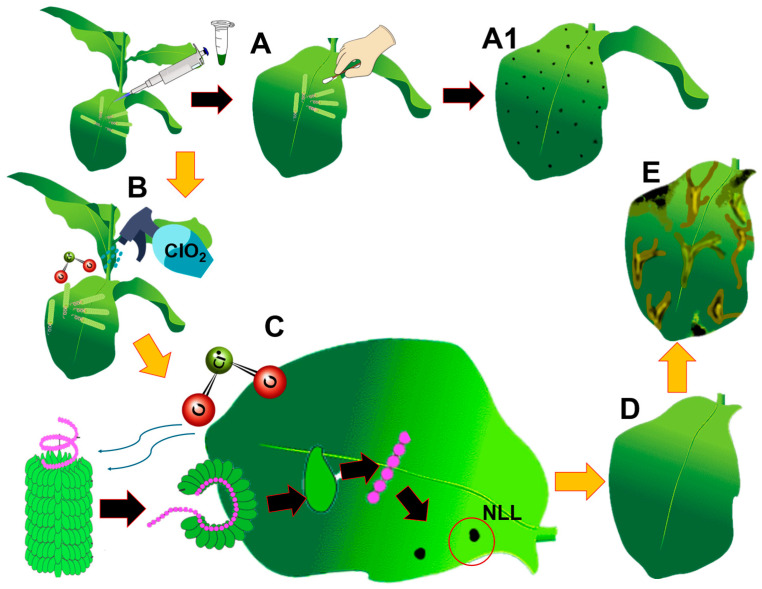
Action of chlorine dioxide on ToBRFV in *Nicotiana longiflora* leaves. A: inoculation of ToBRFV; A1: formation of necrotic local lesions as a response of ToBRFV infection; B: spraying of ClO_2_ 30 min post inoculation; C: action of ClO_2_ on viral particles, capsid protein denaturation and nucleic acid degradation, minimizing the appearance of NLL; D: effectiveness of ClO_2_ at 760 mg L^−1^; E: ClO_2_ greater than 760 mg L-^1^ caused leaf burns due to the accumulation of reactive oxygen species (ROS).

ClO_2_ is an antiviral compound that acts by inhibiting the infection process of ToBRFV viral particles on *Nicotiana longiflora* leaves, reducing the appearance of necrotic local lesions (NLLs), which could act on the capsid protein (PC), degrading the nucleic acids of the virus. In compounds familiar to ClO_2_, rhetorically, they are attributed to the quaternary ammonium salts and ethyl alcohol as “virucidal”; however, in studies carried out in [15,42], they were found not to be effective in preventing the transmission of ToBRFV. Sodium hypochlorite at 3% turned out to be effective, as it prevented the transmission of ToBRFV and did not generate phytotoxicity [15]. However, chlorine was inactivated quickly in the presence of organic matter; therefore, it was not effective for the disinfection of tools and only when applied by spraying did it show good results, which did not prevent the appearance of systemic symptoms. Ref. [42] reported that quaternary ammonium salts at a dose of 0.5 L Ha^−1^ (0.12%) exceeded the manufacturer’s recommendation and did not prevent the onset of NLLs or systemic symptoms in *N. benthamiana*. The mode of action of ClO_2_ depends on the concentration observed on tobacco plants. When concentrations above 760 mg L^−1^ of ClO_2_ were sprayed on *N. longiflora,* this caused phytotoxicity in leaves, but when 760 mg L^−1^ was sprayed, ClO_2_ acted on the proteins and viral genome of ToBRFV, evading the infection in *N. longiflora* (Figure 9).

The sprays with ClO_2_ at 760 mg L^−1^ reduced the spread of ToBRFV during the management of the crop in the open field, and the concentration was measured by OD with the DAS-ELISA technique. In plants located 1.5 m from the inoculum source, chlorine dioxide reduced the spread based on a ToBRFV viral concentration from 38.3 to 51.6%. The transmission of the concentration of viral particles from plant to plant was reduced according to the ClO_2_ sprays ranging from 1.56 to 0.503 compared to untreated plants. This could have been due to the high antioxidant power, capable of degrading the viral RNA, which caused the denaturation of the proteins and the loss of function of the virus [45]. ClO_2_ produced a residual effect after being sprayed on the field-grown tomato plants, impregnated on the cell wall of the leaves, degrading the ToBRFV viral particles and preventing the entry through the openings in the cell wall produced by mechanical damage during the handling of the culture. These authors have reported promising results using chemicals such as hydrochloric acid and sodium hypochlorite. Reference [13] reported that 2% hydrochloric acid (HCl) + 1.5% sodium hypochlorite (NaCl) produced 0% germination at 7 and 14 days after the treatment in seeds for 15 min, whereas 2.5% sodium hypochlorite presented 83.33% germination and completely deactivated ToBRFV. Reference [46] mentions that seeds contaminated with ToBRFV and treated with 2% HCl for 30 min produced 100% disinfection of seeds and improved their quality. It is not new that these compounds showed antiviral activity against ToBRFV; chlorine dioxide is an inorganic compound formed by the acidification of neutral sodium chlorite solutions, which gives the formation of acidified sodium chlorite, and the stabilization of the pH produces the release of a significant amount of chlorine dioxide (ClO_2_) [47].

Reference [15] observed a 25% reduction in infection by ToBRFV in tomato plants (confirmed with ELISA) with the application of Virex, a disinfectant based on quaternary salts; but when tested with Proteacteav, Purell and ethanol/urea/citric acid, none of these were effective in the deactivation of ToBRFV, possibly due to the absence of the lipid membrane, since, for the case of tobamoviruses, they are protected by a capsid, which explains why chemicals with an ethanol base are not effective as disinfectants against ToBRFV. In the experiment evaluated in the greenhouse, tomato plants that were sprayed with ClO_2_ at 760 mg L^−1^ with viral dilutions (1 × 10^−3^ to 1 × 10^−5^) showed a reduced spread of ToBRFV from 75 to 62% (Figure 7). There was no statistical difference in the viral concentration in tomato plants inoculated with different viral dilutions (g of infective tissue per mL); it is possible that the sensitivity of the antibodies used [43] vary depending on whether they are mono- or polyclonal, which affects the detection capacity. The monoclonal antibodies used in this study have a detection limit of 3.86 × 10^−6^ (validation provided by the manufacturer). Ref. [43] found that tomato plants infected with different viral amounts (1 × 10^−3^ to 1 × 10^−5^) induced OD values from 1.5 to 0.6. However, the results in this research, showed viral concentrations from above 2.54 to 1.82; it is possible that this variation was due to the increase in temperature (42 ± 2 °C). Reference [48] took irrigation water samples from different agricultural farms and inoculated them in concentrations of (1:10, *v*/*v*) on tomato plants, which after a DAS-ELISA analysis, witnessed characteristic symptoms of ToBRFV and viral concentrations of 2.65, 2.56 and 2.35 per analyzed sample, compared to 2.67 for the positive control. They also analyzed by DAS-ELISA sap dilutions of tomato plants from 1 × 10^1^ to 1 × 10^−12^, which expressed ODs from 3.33 to 0.01; however, when inoculated in tomato plants, they witnessed optical densities from 2.02 to 0.1, coinciding relatively with the results presented in Figure 5 and Figure 6. This also validates the results obtained by the authors of [43], who reported that infected plants with a dilution of 1 × 10^−6^ perform as asymptomatic, which affects the agronomic parameters of the studied tomato plants.

**Figure 10:**
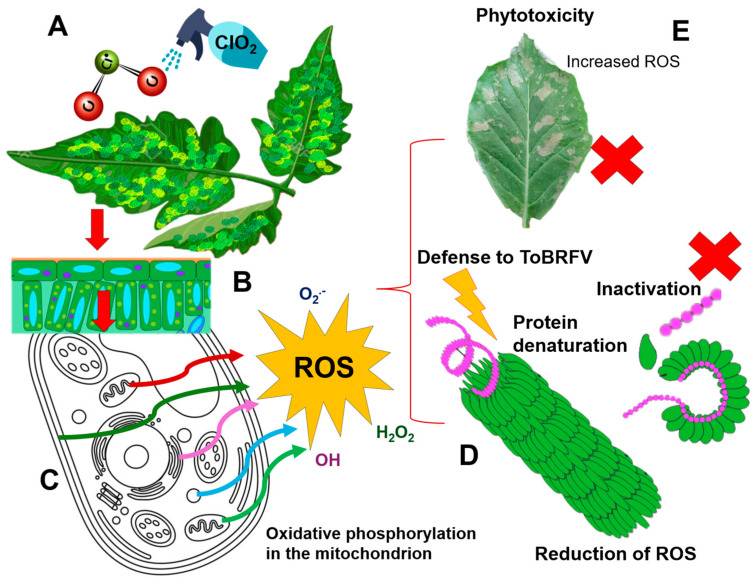
Action of ClO_2_ on the replication of tomato plants grown in greenhouse. (**A**) ClO_2_ spraying at 760 mg L^−1^, one day after inoculation the viral particles are replicating in the host; (**B**) ClO_2_ penetrates through the cell wall of the plant, reaching the plant cells. (**C**) The entry of ClO_2_ reduces oxygen, promotes aerobic metabolism and causes the production of ROS compounds (O_2_^.−^, H_2_O_2_, HO^•−^); (**D**) ROS act by denaturing viral proteins and degrading ToBRFV nucleic acids; (**E**) the accumulation of ROS causes phytotoxicity in the leaves.

In an assay by [7], it is mentioned that inhibition in tobacco mosaic virus (TMV) was greater than 400 mg L^−1^, 48 h post inoculation (hpi), with a reduction in viral concentration of 84.9%, whereas ClO_2_ at 200 mg L^−1^ produced a reduction of 54.1%. In other studies, [49] mentioned that the infectious transmission of ToBRFV in plants mediated by soil and incubated with 3% Cl-TSP + 1% KOH showed an infectivity of 6.3%, compared to the untreated control (39.7%). Deoxidation generates reactive oxygen species (ROS) [50]. Ref. [51] described that ROS is produced from the aerobic metabolism of plants by using oxygen as the final electron acceptor. Oxygen when reduced is produced in singlet activated form (^1^O_2_), and also by the transfer of one or three electrons, the superoxide radical (O_2_^.−^), hydrogen peroxide (H_2_O_2_) or the hydroxyl radical (HO^•−^) are formed, which are molecules formed because of cellular metabolism. Within plants, ROS is continuously produced in different cellular organelles such as: mitochondria, chloroplasts, peroxisomes, endoplasmic reticulum and in the plasma membrane [52] where the production of ROS has been established as one of the first signaling events involved in the response to biotic and abiotic stress [53,54]. The production of ROS acts as activation of enzymatic defense mechanisms, such as lipid peroxidation [55]. Excessive amounts of ROS can produce phytotoxicity in tomato plants, interfering in biochemical reactions and reducing the chlorophyll content, which affects photosynthesis [56,57]. It is possible that these aspects are related to the effect of ClO_2_ on the replication of ToBRFV in tomato plants grown in a greenhouse, so it is possible that chlorine dioxide acts directly by exciting the cellular organelles to increase the production of ROS that will act directly with the viral particles, denaturing the proteins and degrading the nucleic acid of ToBRFV, although an excessive accumulation of ROS could cause cellular disruptions, which is related to phytotoxicity in the leaves. Higher concentrations of ClO_2_ (760 mg L^−1^) caused burns in *N. longiflora* leaves and reduced ToBRFV replication based on viral concentration (Figure 10).

Viral load quantification has been used for the evaluation of disinfectants and antiviral compounds for the alternative management of ToBRFV. Refs. [39,58] used experimental plants to propagate ToBRFV, without knowing the viral concentration of the inoculum. In other studies, ELISA has been used as a tool for virus quantification in plant samples [18]. In some studies, the exact amount of the viral load has been unknown, which affected the type of antibody used, and this, in turn, produced variations in viral concentrations in different species and their hosts [59]. Ref. [40] showed that the viral concentration in the inoculum obtained from *N. benthamiana* and *N. clevelandii* varied according to the host. They commented that before quantification, for the effectiveness of the evaluated chemical, it is necessary to determine the initial viral load before the treatments, and then the determination of the remaining load. The evaluation of different viral dilutions of ToBRFV and the effect on agronomic parameters of the plant contribute to the decision making for the management of the virus. Ref. [60] mention that ToBRFV caused a negative effect on the quality of the fruit, size and color, and those plants contaminated with ToBRFV through open field management and inoculated with different viral dilutions formed fruits with severe symptoms of roughness, mottling and irregular ripening, reducing the quality, size, number and uniformity. Refs. [22,32,61] determined the impact of ToBRFV on plant height, stem diameter, root and plant dry weight, where they found a reduction in these variables.

The results coincided with those obtained in this research, where plants inoculated with ToBRFV reduced by 21.87% in terms of SD and the PH decreased by 17.04%; on the other hand, the PDW decreased by 53.88%, followed by 19.87% in RDW. Results showed that ClO_2_ increased the number of flowers from 19.67 to 40.77% in open-field plants and in the greenhouse by 4.03%. The NFR increased from 32 to 38% in the open field and in the greenhouse, it was improved from 10 to 53%. Yield increased with chlorine dioxide spraying from 38 to 34% as compared to plants located at the beginning of each furrow (IS) in the open field, and in the greenhouse from 47 to 45%. These results coincided with losses reported that ranged from 12.9 to 55% [3,28,61,62]. The prediction model of yield loss was adjusted to the linear model $\left(y=0.2496\left(x\right)+0.0234,{\;R}^{2}=0.97\right)$ that will allow the estimation of yield losses 50 days post ToBRFV infection, using the viral concentration in the plant under open-field conditions. Moreover, it could be used in a practical way to know the impact of the virus on tomato yield grown nationally and, based on this, to propose phytosanitary and regulatory strategies, which will reduce the spread of ToBRFV.

The need to quantify the progress of the accumulated damage from epidemics affecting important crops has become relevant since the emergence of pathogenic diseases. Plant viruses have been characterized as causing uncontrollable epidemics due to the lack of control methods. Ref. [37] mentioned that the fundamental way to represent a plant disease epidemic is to trace the level of the disease at various times or distances. He called this modeling the “disease progress curve”, where they summarize the host–pathogen interaction and the environment in which the disease develops. Ref. [63] reported that the progress of ToBRFV follows a sigmoid logistic model, starting with an initial phase where there is a slow increase in incidence, then the exponential growth phase and, finally, the maximum number of infected plants, better known as the deceleration phase. Such a model behaved similarly in the present evaluation of tomato plants grown in a field and greenhouse (Table 4 and Table 5). Ref. [64] evaluated 0.5 Ha of tomatoes grown in greenhouse conditions for a nine-month period and showed an accelerated increase in the transmission spread of ToBRFV from 5.8 to 80% in just three months. This shows that the main mean of transmission of ToBRFV is through plants since it only needs a few plants to reach 100% incidence. This means that the best method to control the spread of the virus lies in reducing the viral load in infected plants and applying control methods to inhibit mechanical transmission during routine crop handling, hence the importance of using antiviral compounds that inhibit mechanical transmission and reduce ToBRFV replication. Disinfectants can be corrosive and phytotoxic for the management of tomato plants because most of them have been used for the disinfection of seeds, tools, substrates and surfaces [13,15,16,17,18,19], which makes it difficult to scale them in greenhouse and open-field crops. The use of environmentally friendly compounds to control emerging viruses is currently a challenge in terms of sustainable development [14]. Chlorine dioxide can be an effective and friendly alternative to manage the epidemic caused by the species *Tobamovirus fructirugosum* (ToBRFV). Thus far there are few studies available where the specific evaluation of the damage caused by ToBRFV in tomato production, the spraying of a chemical compound that reduces the impact of ToBRFV on tomato plants grown in the field and greenhouse and the estimation of losses in the production of tomato yield are mentioned.

## 5. Conclusions

ClO_2_ at 760 mg L^−1^ does not produce phytotoxicity in *Nicotiana longiflora* leaves but reduces the transmission of ToBRFV from the inoculum source by 60%, and decreases the spread of ToBRFV by 48% (open field) and 81% (greenhouse). ClO_2_ spraying improves the agronomic parameters of tomato plants and reduces yield losses by 48% in the open field and 85% in the greenhouse. The existence of a linear relationship [0.2496 (x) + 0.0234] between the viral concentration (OD ELISA) and the yield losses caused by ToBRFV is demonstrated. The quantification of the AUCDP caused by ToBRFV was reduced according to the ClO_2_ sprays in field and greenhouse plants.

## Figures and Tables

**Table 1:** Models evaluated for yield loss estimation.

Models	Equation	Linearized Equation	Authors
Berger	$y=\left(\frac{1}{\left[1+exp(-\ln\;[\frac{y_{0}}{1-y_{0}}])+r(pr)\right]}\right)$	$ln[\left(\frac{y}{1-y}\right)=\left(\frac{Y_{0}}{1-y0}\right)\;r\left(pr\right)]$	[34,35]
inverted exponential	$y=y_{0}\exp({pr-r}_{E})$	$ln\left(y\right)={ln(y}_{0})-r[ln\left(pr\right)]$	[36,37]
Exponential model	$y=y_{0}\exp[r_{E}(pr)]$	$ln\left(y\right)={ln(y}_{0})+r_{E}(pr)$	[37]
Monomolecular	$y={1-[(1-(-y}_{0})(\exp-r_{E}\left(pr)\right)]$	$ln\left(\frac{y}{1-y}\right)=ln\left(\frac{Y_{0}}{1-y0}\right)+r_{M}(pr)$	
Logistics	$Yt\left(\frac{1}{1+\exp[-ln[(\frac{y_{0}}{1-y_{0}}+r(pr))]}\right)$	$ln\left(\frac{y}{1-y}\right)=ln\left(\frac{Y_{0}}{1-y0}\right)+r_{L}\;pr$	
Line	$y=y_{0}+r\;(x)$	-	[38]

$\ln\;[\frac{y_{0}}{1-y_{0}}]:$ intersection of the axis; $y_{0}$: integration constant (initial level of the disease); r logarithmic rate of increase in disease, better known as average rate of disease, logistic rate or epidemic rate; pr: yield losses; y: spread of the disease; exp: base exponential constant of the natural logarithms = 2.7183; d: transmission distance; 1−y: proportion of plant tissue available to the pathogen; $r_{E}$: absolute rate of change and relative to the level of y; $r_{M}$: slope of the constant line; $r_{L}$: efficiency of the inoculum in an r logistical; x: viral load (OD ELISA).

**Table 2:** Effective concentration of ClO_2_ that reduced necrotic local lesions in *Nicotiana longiflora* leaves.

Treatments	Phytotoxicity ^A^	NLLs ^B^
100 mg L^−1^ ClO_2_ + ToBRFV	0.00 ± 0.00 a	29.28 ± 0.42 d
250 mg L^−1^ ClO_2_ + ToBRFV	0.00 ± 0.00 a	19.61 ± 3.10 c
500 mg L^−1^ ClO_2_ + ToBRFV	0.00 ± 0.00 a	9.76 ± 0.34 b
760 mg L^−1^ ClO_2_ + ToBRFV	0.00 ± 0.00 a	1.53 ± 0.07 a
1520 mg L^−1^ ClO_2_ + ToBRFV	17.44 ± 0.82 b	0.74 ± 0.09 a
3040 mg L^−1^ ClO_2_ + ToBRFV	26.55 ± 1.41 c	0.36 ± 0.07 a
6080 mg L^−1^ ClO_2_ + ToBRFV	44.31 ± 0.85 d	0.00 ± 0.00 a
Distilled H_2_O (1 × 10^0^)	0.00 ± 0.00 a	0.00 ± 0.00 a
Positive control	0.00 ± 0.00 a	38.13 ± 4.81 e
6080 mg L^−1^ ClO_2_	43.68 ± 1.33 d	0.00 ± 0.00 a

NLLs: necrotic local lesions; positive control: inoculum ToBRFV at 1:10 (p/v); ^A^ arcosene of phytotoxicity; ^B^ NLL formation for each 100 µL^−1^ of infectious wisdom. Stockings with a common letter are not significantly different (*p* > 0.01).

**Table 3:** Area under the ToBRFV disease progression curve.

	AUCDP	
Distance	ClO_2_	Control
IS	310.67 ± 18.23 j	374.09 ± 14.59 l
1.5	102.77 ± 22.47 e	369.09 ± 14.45 l
3	94.54 ± 18.42 e	358.50± 14.09 kl
4.5	84.95 ± 18.61 de	338.42 ± 13.61 k
6	69.05 ± 17.42 cd	282.15 ± 13.30 i
7.5	55.35 ± 12.69 bc	255.68 ± 1204 h
9	45.58 ± 10.42 ab	247.02 ± 11.82 h
10.5	40.97 ± 10.08 ab	243.57 ± 11.45 h
12	38.62 ± 8.87 ab	209.54 ± 11.50 g
13.5	32.75 ± 9.69 a	192.47 ± 10.87 fg
15	26.62 ± 10.09 a	181.66 ± 9.74 f
NC	0.00 ± 0.00 a	0.00 ± 0.00 a

ClO_2_: dilutions treated with chlorine dioxide; Control: untreated dilutions; NC: negative control; IS: plants located at the beginning of each furrow and inoculated with 100% infective sage; dpi: days post inoculation. Means with a common letter are not significantly different (*p* > 0.01).

**Table 4:** Adjustment of a model for estimating yield losses in field plants.

Models	Equation	Substituted Equation	R^2^
Berger	$y=\left(\frac{1}{\left[1+exp(-\ln\;[\frac{y_{0}}{1-y_{0}}])+r(pr)\right]}\right)$	$y=\left(\frac{1}{\left[1+exp(-\ln\;[\frac{0.045}{1-0.045}])+0.075(d)\right]}\right)$	0.94
Inverted exponential	$y=y_{0}\exp({pr-r}_{E})$	y=0.6644exp⁡(pr−0.0436)	0.89
Exponential	$y=y_{0}\exp[r_{E}(d)$]	y=0.043exp⁡[0.91 (pr)]	0.68
Monomolecular	$y={1-[(1-(-y}_{0})(\exp-r_{E}(pr))]$	y=1−[1−(−0.24)(exp−⁡0.073(pr))]	0.80
Logistic	$yt=\left(\frac{1}{1+\exp[-ln[(\frac{y_{0}}{1-y_{0}})+r(pr)]}\right)$	Y = $yt=\left(\frac{1}{1+\exp[-ln[(\frac{-0.0072}{1-0.0072})+0.4838(pr)]}\right)$	0.81
Lineal	$y=y_{0}+r\;(x)$	y=0.0234+0.2496 (D0)	0.97

R^2^: Completion coefficient; $\ln\;[\frac{y_{0}}{1-y_{0}}]:$ intersection of the axis; $y_{0}$: integration constant (initial level of the disease); r: logarithmic rate of increase in the disease, better known as logistic rate or epidemic rate; pr: losses in performance; y: spread of the disease; exp: the exponential constant of the natural logarithms = 2.7183; d: transmission distance; 1−y: proportion of plant tissue available for the pathogen; y_t_: is the amount of illness over time (t); $r_{E}$: absolute rate of change and relative to the level of y; $r_{M}$: slope of the constant line; $r_{L}$: inoculum efficiency in a logistic r; (x): viral concentration (DO ELISA); D0: viral concentration DO ELISA.

**Table 5:** Area under the disease progress curve (AUCDP) in tomato plants inoculated with ToBRFV at 50 dpi in greenhouse conditions.

	AUCDP	
Treatments	ClO_2_	Control
NC 1 × 10^0^	0.00 ± 0.00 a	0.00 ± 0.00 a
1 × 10^−3^	105.26 ± 1.45 g	220.91 ± 4.16 j
1 × 10^−3.5^	78.80 ± 1.75 e	167.64 ± 4.1 i
1 × 10^−4^	56.93 ± 1.88 d	122.77 ± 4.63 h
1 × 10^−4.5^	43.32 ± 2.60 c	94.92 ± 5.54 f
1 × 10^−5^	34.86 ± 1.88 b	76.35 ± 2.30 e
PC 1 × 10^1^	309.10 ± 6.01 k	309.10 ± 6.01 k

dpi: days after inoculation. ClO_2_: plants infected with different viral dilutions and treated with chlorine dioxide; Control: plants infected only with viral dilutions of inoculum; NC: negative control; PC: positive control. Means with a common letter are not significantly different (*p* > 0.01).

## Data Availability

The underlying data of this manuscript are available upon reasonable request from the authors.
